# Complete chloroplast genome of green alga *Caulerpa sertularioides f. longipes* (J.Agardh) Collins, 1909

**DOI:** 10.1080/23802359.2019.1676668

**Published:** 2019-10-15

**Authors:** Rongxia Wang, Hongtao Liu, Yongbo Wang, Hongji Ke, Jiawei Fan, Fuxiao Chen, Wei Tan

**Affiliations:** Hainan Provincial Engineering Research Center for Tropical Sea-Farming, Hainan Academy of Ocean and Fisheries Sciences, Haikou, PR China

**Keywords:** Chloroplast genome, *Caulerpa sertularioides f. longipes*, phylogenetic analysis

## Abstract

The complete chloroplast (cp) genome of *Caulerpa sertularioides f. longipes* was first assembled and characterized using Illumina pair-end sequencing from the South China Sea. It is 133,626 bp long, with a GC content of 32.94%. In total, 92 genes were identified in the genome, and they consisted 60 protein-coding genes (PCGs), 30 tRNA genes, and 2 rRNA genes. Like other species in *Caulerpa*, the whole cp genome of *C. sertularioides f. longipes* did not demonstrate an obvious quadripartite structure. A total of 31 microsatellites (SSRs) were identified in the cp genome using MISA. A phylogenetic tree revealed that *C. sertularioides f. longipes* was closer to *Caulerpa cupressoides*, which further clarified the phylogenetic relationships of species in *Caulerpa*.

*Caulerpa sertularioides f. longipes* is one forma of *C. sertularioides*, known as green feather algae, which can be confused with *Caulerpa taxifolia*, a highly invasive algae (Fernández and Cortés 2005). With great ornamental value, it is sought after by aquarium enthusiasts. In Western-Pacific region, it is mainly distributed in the South China Sea. In this algae, plenty of polysaccharides and sterols are found that could be an ideal natural bait for fish (Shevchenko et al. 2009). It has also been reported that extracts from this algae can elicit pronounced antinociceptive and anti-inflamatory activity (Brito da Matta et al. 2011), antibacterial, and antivirus activity (Zandi et al., 2006; Darah et al. 2014; Esquer-Miranda et al. 2016). In addition, it is useful in aquaculture wastewater treatment and circulating water systems due to its high efficiency of absorbing inorganic nitrogen (Gao et al. 2016; Zheng et al. 2016).

The fresh thallus of *C. sertularioides f. longipes* was obtained from Paracel islands (N16°49′58.83″, E112°20′1.86″) in the South China Sea. The voucher specimens (2012XS001) were stored in Marine Biological Specimen Room, Hainan Provincial Marine Science, and Technology Museum. The samples were used for the total genomic DNA extraction with the modified CTAB method. The whole chloroplast (cp) genome was conducted with 150 bp pair-end reads on the Illumina Hiseq Platform, assembled with NOVOPlasty and annotated based on NCBI cp database comparison. The annotations of cp genome were submitted to GenBank database under Accession (No. MK792750). The phylogenetic analysis was carried out based on super matrix of 21 protein-coding genes (PCGs) in 9 cp genomes of species in Caulerpaceae, using maximum-likelihood (ML) method with 1000 bootstrap replicates.

The complete cp genome of *C. sertularioides f. longipes* was 133,626 bp in length. The nucleotide base content of A, G, T, and C was 33.30%, 16.71%, 33.76%, and 16.23%, respectively, with the overall AT content of 67.06%. Ninety two genes were identified in the genome including 60 PCGs, 30 tRNA genes, and two rRNA genes. Like other species in *Caulerpa*, the cp genome of *C. sertularioides f. longipes* did not demonstrate a quadripartite structure and lacked the large rRNA operon-encoding inverted repeat (IR). About 43 PCGs, 18 tRNA genes, and two rRNA genes were encoded in the forward strand, 17 PCGs and 12 tRNA genes were encoded in the reverse strand. 16 s rRNA gene was interrupted by a large intron into two fragments. Additionally, a total of 31 microsatellites (simple sequence repeats [SSRs]) were identified in the *C. sertularioides f. longipes* cp genome using MISA. All of these SSRs were mononucleotides, most of the repeats were A/T except one was (G)_11_.

The results of the phylogenetic tree (Figure 1) revealed that *C. sertularioides f. longipes* was closer to *Caulerpa cupressoides* which was first clustered with *Caulerpa manorensis*, *Caulerpa okamurae*, and *Caulerpa racemose*. The data of *C. sertularioides f. longipes* cp genome will provide new evident for further identification of intraspecific forma and variety, and to clarify the phylogeny and evolution of green alga Bryopsidales.

## Figures and Tables

**Figure 1.**
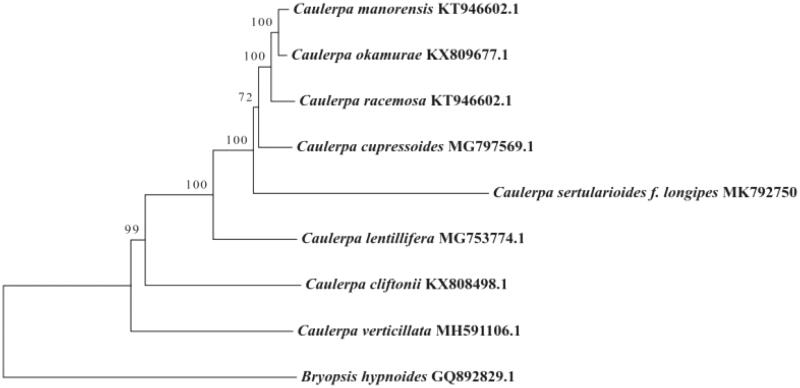
Phylogenetic tree of 9 species based on 21 PCGs from chloroplast genomes in *Caulerpaceae* by maximum-likelihood (ML) method. *Bryopsis hypnoides* was used as an outgroup.
